# Characteristics and Yield of Modern Approaches for the Diagnosis of Genetic Causes of Kidney Stone Disease

**DOI:** 10.3390/genes15111470

**Published:** 2024-11-14

**Authors:** Andrea Spasiano, Mirko Treccani, Elisa De Tomi, Giovanni Malerba, Giovanni Gambaro, Pietro Manuel Ferraro

**Affiliations:** 1 Section of Nephrology, Department of Medicine, Università degli Studi di Verona, Piazzale L.A. Scuro 10, 37134 Verona, Italy; andrea.spasiano@univr.it (A.S.); elisa.detomi@univr.it (E.D.T.); giovanni.gambaro@univr.it (G.G.); 2 Department of Neurosciences, Biomedicine and Movement Sciences, Università degli Studi di Verona, Piazzale L.A. Scuro 10, 37134 Verona, Italy; mirko.treccani@univr.it; 3 GM Lab, Department of Surgery, Dentistry, Paediatrics and Gynaecology, Università degli Studi di Verona, Piazzale L.A. Scuro 10, 37134 Verona, Italy; giovanni.malerba@univr.it

**Keywords:** genetics, nephrocalcinosis, nephrolithiasis, kidney stone disease, whole-exome sequencing

## Abstract

Background: Kidney stone disease (KSD) is characterized by an increasing prevalence worldwide, representing an important clinical issue and a financial burden for healthcare systems. A KSD-causing monogenic variant is traditionally expected in up to 30% of children and 1–5% of adults forming stones, confirmed by a strong connection between a positive family history and KSD. The insufficient use of genetic testing in these patients is associated with a lack of perceived benefit and a scarce awareness of inherited kidney diseases. Genetic testing has important practical implications, such as the possibility of earlier diagnoses, familial counseling, and tailored therapy, based on the evaluation of fine-mapped pathogenic variants. Our aim is to analyze the current evidence on genetic testing in KSD patients to whom genetic tests were applied without strict a priori selection criteria, to provide an overview of its diagnostic yield and factors potentially affecting it (such as the age of KSD onset, a familial history of KSD, consanguinity, and extrarenal features). Methods: A literature review was performed, selecting original articles published in the last 10 years concerning genetic investigations in patients affected by nephrolithiasis or nephrocalcinosis. Available data were subsequently extracted and analyzed. Results: In total, 13 studies on 1675 patients (77% pediatric populations) were included; 333 patients were determined to be affected by a monogenic disorder, with an overall yield of about 20%. The likelihood of a positive genetic finding was much higher in pediatric (26%) than adult populations (8%). Cystinuria was the most common diagnosis in both populations. After the removal of conditions that could be identified with a stone composition analysis or urinary chemistry investigation, the diagnostic yield dropped to 19% among pediatric patients and below 5% for adults. Conclusions: Genetic testing should be considered in KSD pediatric patients and in selected subgroups of adults with suggestive features when a diagnosis is not established after stone examination and blood as well as urine metabolic profiling.

## 1. Introduction

Kidney stone disease (KSD) is a condition characterized by an increasing prevalence across the world in recent decades [1,2], as well as high recurrence rates [3], representing a clinical as well as a financial burden for healthcare systems, with a projected increase in the cost of KSD of an additional USD 1.24 billion per year by 2030 [4]. This is likely a consequence of the modern lifestyle and diet of high-income countries [5]. Concurrently, while the rising incidence of KSD in developed countries reflects the increased consumption of salt and protein, along with the spread of obesity, diabetes, and metabolic syndrome [6,7,8], the increase in developing countries is likely due to malnutrition and water deprivation [9]. In recent years, there has been a significant increase in KSD prevalence among women, narrowing the gender gap (9.4% vs. 11.9% of men in 2018) [10], not only depending on discrepancies in acid–base handling, calcium tubular reabsorption, hormonal changes, and genetics, but also because of dietary and lifestyle changes in women over the last decades [11,12].

KSD can be associated with different etiologies: a KSD-causing monogenic variant is traditionally expected in up to 30% of children and 1–5% of adults forming stones [13], with a large number of genes involved; at the same time, polymorphisms in a series of genes have been implicated in the tubular control of stones’ constituents and crystallization’s inhibitors in several genome-wide association studies [14,15,16,17,18]. Confirmatory is the remarkable link between a positive history of KSD in families and blood relatives, and in monozygotic twins with a 30–50% higher risk of stone recurrence, an increased severity of KSD, and an earlier age of onset in patients with a positive family history [19,20,21,22]. Given the close association between KSD and chronic kidney disease (CKD) [23], with a higher risk of kidney failure compared with non-stone formers [24,25,26], a precise diagnosis of the underlying cause of KSD is essential for personalized treatment and to improve patient outcomes.

Unfortunately, many patients and their physicians are unaware of the possibility of a genetic origin of their kidney disorder. Moreover, the insufficient use of genetic testing in these patients is associated with a lack of perceived benefit, scarce awareness of inherited kidney diseases, difficulties in interpreting the identified genetic variants, high costs, and poor availability or accessibility to tests [27,28,29]. The resulting ambiguity in diagnosis negatively impacts clinical management, particularly given the availability of targeted therapies for certain specific monogenic forms of KSD, such as cystinuria and primary hyperoxaluria [30,31,32].

Nowadays, high-throughput sequencing techniques, also known as next-generation sequencing (NGS), offer significantly improved opportunities for accurate genetic diagnosis compared with existing Sanger sequencing. As a matter of fact, NGS allows for the simultaneous sequencing of the following:

- Exons of a subset of known pathogenic genes or candidate genes associated with a specific disease, called targeted sequencing;
- Exons of all protein-coding human genes (about 20,000), known as whole-exome sequencing (WES), frequently applied in family studies or in strongly suspicious cases with a negative genetic test, to overcome the limitations of panel sequencing;
- The complete genome, named whole-genome sequencing (WGS) [33].

Currently, WES stands out as the favored diagnostic tool thanks to its ability to test most KSD-associated genes, pointing out small variants in the screened genes.

On the other hand, WGS can theoretically detect large copy number variations (CNVs) and variants in complex genomic regions (such as introns and promoters), but it is rarely used as a screening tool because of costs, time constraints, and the complex interpretation of non-coding variants [28].

The main goal of this study is to analyze the current evidence on genetic testing in patients affected by KSD to whom genetic tests were applied without strict a priori selection criteria, to provide an overview of its diagnostic yield and factors potentially affecting it, as well as to establish its possible future application to guide the correct diagnosis and clinical management of these patients, consequently leading to the best therapeutic options.

## 2. Materials and Methods

We performed a literature review, analyzing several studies concerning genetic investigations in patients affected by nephrolithiasis or nephrocalcinosis, mainly focusing on different genetic testing methods and their diagnostic yield. The PubMed database was searched up to April 2024, using the following set of terms:

(“urolithiasis”[MeSH Terms] OR “nephrocalcinosis*”[MeSH Terms]) AND
(“genetic testing”[MeSH Terms] OR “exome sequencing”[MeSH Terms] OR “MPS”[Title/Abstract] OR “massively parallel sequencing”[Title/Abstract] OR “next generation sequencing”[Title/Abstract] OR “gene*”[Title]).

All of the original articles selected were published in the last 10 years, considering only studies on human subjects; since we were interested in unbiased approaches to genetic investigation, the candidate gene approach with a case–control design was not considered.

We mainly focused on patients’ characteristics that can potentially influence the diagnostic yield, such as the degree of consanguinity, the percentage of patients with a positive family history, the percentage of KSD presenting with extrarenal features, the mean age of KSD onset, and the percentage of patients with kidney failure. We tried to assess how these characteristics positively impacted the reported diagnostic yield in different studies and if they were associated with a higher probability of a positive genetic test in KSD patients. Moreover, we extrapolated the characteristics of the genetic assay and the number of analyzed genes to evaluate the correlation between the extent of the genetic analysis (targeted gene, exome, or genome sequencing) and the subsequent diagnostic yield.

## 3. Results

Our search strategy identified 346 papers. After further selection, with the exclusion of non-pertinent articles, we included 11 works for detailed evaluation. Another five papers were included via backward snowballing.

Three studies (Huang et al. [34], Cogal et al. [35], and Anderegg et al. [36]) were excluded due to a possible selection bias, i.e., a strong suspicion of a Mendelian form of KSD and a previous Sanger sequencing evaluation in the Cogal et al. study.

Our final analysis included 13 studies on 1675 patients in whom genetic tests were carried out without any strict selection criteria. The key characteristics are reported in Table 1.

Seven studies (54%) were performed with a WES approach, and six (46%) with a targeted panel approach. The majority of the studies (*n* = 10, 77%) were restricted to pediatric/young populations (with two studies extending this definition to individuals ≤ 21 and 25 years old, respectively), whereas two studies (15%) were performed on mixed populations and one (8%) was restricted to adults.

In the studies performed on mixed populations, the percentage of pediatric patients was 31% and 39%, respectively. So, overall, genetic investigations in adult patients were relatively limited.

Nephrocalcinosis was often absent or not reported; in those studies reporting it (*n* = 5, 38%), the prevalence of nephrocalcinosis ranged between 10 and 51% of the samples, either isolated or combined with nephrolithiasis. Family history, reported in six studies, ranged from 18 to 58%. Instead, data about consanguinity and concurrent chronic kidney disease are fragmented and limited, with only five and four studies, respectively, analyzing these features, not allowing the diagnostic yield to be evaluated in these specific subgroups.

Of the 1675 participants, 333 were found to be affected by a monogenic disorder, leading to an overall yield of about 20%. The average yield in studies performed with a targeted panel approach was 16%, significantly lower than in studies implementing an exome sequencing approach (31%).

The likelihood of a positive genetic finding was much higher in pediatric (26%, range of 11 to 46%) compared with adult populations (8%, range of 7 to 11%). The individual diagnoses are reported in Table 2.

**Figure 1:**
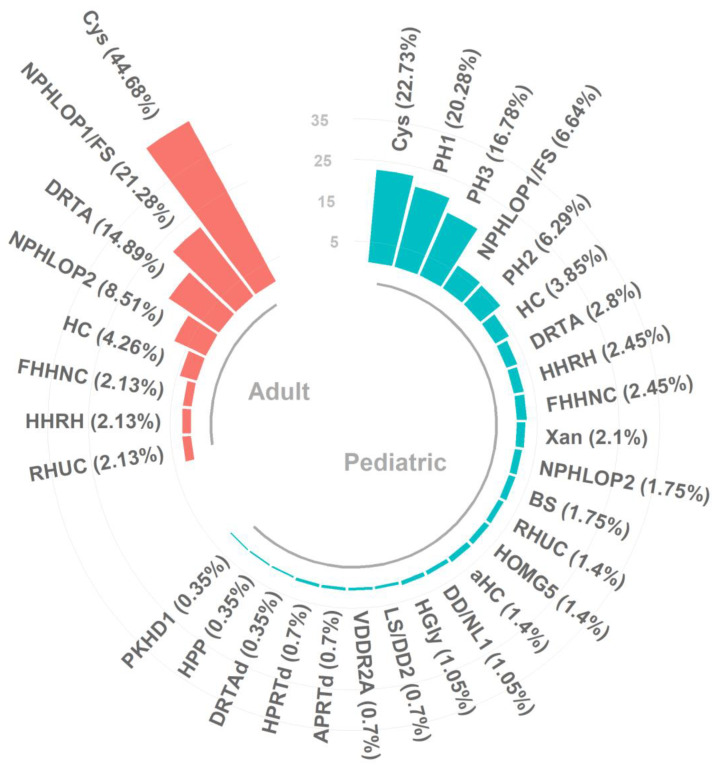
Distributions of monogenic conditions across pediatric and adult populations in the included studies. Cys, cystinuria; HC, hypercalciuria; RHUC, renal hypouricemia; dRTA, primary distal renal tubular acidosis; DD/NL1, Dent disease/nephrolithiasis type 1; HHRH, hereditary hypophosophatemic rickets with hypercalciuria; FHHNC, familial hypomagnesemia with hypercalciuria and nephrocalcinosis; HOMG5, hypomagnesemia 5, renal, with ocular involvement; BS, Bartter syndrome; PH1, primary hyperoxaluria 1; LS/DD2, Lowe syndrome/Dent disease 2; aHC, absorptive hypercalciuria; DRTAd, renal tubular acidosis with deafness; PH2, primary hyperoxaluria 2; APRTd, adenine phosphoribosyltransferase deficit; HGly, hyperglycinuria; Xan, xanthinuria; HPP, hypophosphatasia; HPRTd, hypoxanthine guanine phosphoribosyltransferase deficiency; PKHD1, autosomal recessive polycystic kidney disease; PH3, primary hyperoxaluria 3; VDDR2A, vitamin D-dependent rickets, type 2A; NPHLOP1/FS, hypophosphatemic nephrolithiasis/osteoporosis/Fanconi syndrome; and NPHLOP2, hypophosphatemic nephrolithiasis/osteoporosis-2. Pediatric data are in sky blue, adult data in red.

Overall, cystinuria was the most common diagnosis in both pediatric and adult populations (23% and 45% of all genetic diagnoses, respectively); primary hyperoxaluria type 1 (20%) and type 3 (17%) were the other most frequent diagnoses among pediatric patients, whereas hypophosphatemic nephrolithiasis (21%) and primary distal renal tubular acidosis (dRTA) (15%) were most common among adults (Figure 1).

Of note, the genetic yield after the removal of conditions that could be identified with an appropriate stone composition analysis or urinary chemistry investigation (cystinuria, xanthinuria, and dRTA) dropped to 19% among pediatric patients and below 5% (4.7%) for adults.

Specifically, until 2015, the application of genetic testing in patients with KSD was sporadic due to high costs and difficult interpretation. Studies were time-consuming and often limited to Sanger-sequencing-based methods and all-exon PCR-based assays of a few specific genes in families of patients with extremely rare syndromes [37,38,39,40].

With the introduction of NGS, the potential for achieving a specific genetic diagnosis in KSD has increased. Halbritter et al. [41] reported the first broad genetic evaluation of KSD-causing genes, enrolling 272 pediatric and adult patients affected by KSD of unknown origin. The authors detected 50 likely pathogenetic mutations in 14 genes, with an overall diagnostic yield of 14.9%, finding a putative variant in 20.8% of individuals with an onset < 18 years, while in adult patients a causative mutation was detected only in 11.4% of cases. Altogether, genetic diagnoses provided additional information and led to important practical implications in about 40% of patients. Nonetheless, the most frequent KSD-causing gene was determined to be SLC7A9, associated with cystinuria, whose diagnosis is typically achieved through a detailed urinary metabolic study and thorough stone analysis.

The same group focused its attention on a pediatric population, confirming the results of the previous study with a molecular diagnosis in 16.8% of patients [42]. In this study, the authors analyzed potential correlations between sex and monogenic causes but found no significant differences. Nevertheless, all of the patients included in these two studies came from specialized kidney stone clinics at international tertiary care centers, potentially introducing selection bias.

This avenue of investigation continued, expanding to include other international stone centers and advancing beyond panel sequencing. Daga et al. [43] reported the results of WES in 51 families with at least one episode of a kidney stone or ultrasound evidence of nephrocalcinosis before 25 years of age, reaching a high diagnostic rate (29.4%). A putative mutation was found in 25% of patients with nephrolithiasis and in 44.8% of those affected by nephrocalcinosis. For the first time, the authors conducted an in-depth analysis of factors that could potentially impact diagnostic yield. They observed a lower median age of onset in patients with a monogenic cause, not finding causative mutations in patients with an age of onset > 15 years. Furthermore, the genetic test turned out to be positive in 41% of families with several cases of KSD, confirming the high rate of a monogenic cause in familial cases. Authors also stressed the higher detection rate in patients from regions with an elevated degree of consanguinity (such as the Middle East and Egypt), with a monogenic cause detected in 75% of consanguineous families. However, the mutation detection rate in individuals without a family history, consanguinity, or at a younger age of onset was also significant, highlighting the importance of genetic testing in KSD patients, even in the absence of these “alarm signals”.

These studies inspired other researchers to explore the genetics of KSD in cohorts of different ethnicities. In their study on a population from Pakistan, Amar et al. [44] reported an overall diagnostic yield of 8.5% of cases. The lower detection rate of monogenic causes was attributed to the likely involvement of yet-unidentified genes or the relatively limited coverage of exon sequencing, which cannot detect CNVs. Nonetheless, the high frequency of monogenic mutations in patients with a family history of KSD (12%) and an earlier age of onset (11% in patients aged 1–20 years vs. 0% in those aged 61–80 years) was confirmed.

Successively, Fang et al. [45] demonstrated a diagnostic yield of 29.3% in a cohort of 2256 Chinese pediatric patients with kidney and urological diseases. WES improved diagnostic precision, as KSD was diagnosed in only 3.6% of cases during the primary clinical evaluation. A higher detection rate in patients with a positive family history or consanguinity was observed, confirming previous findings.

Indeed, Zhao et al. [46] reached a higher molecular diagnostic yield (36%) with 100% conformity between molecular diagnoses and metabolic evaluation. Nonetheless, only severe pediatric KSD patients were included and about 47% of patients refused WES in the enrollment process, potentially biasing the real estimate of diagnostic rate. These results were similar to those of Ziyadov et al. [47] who achieved an overall detection rate of 37.5%. The data confirmed a correlation between familiarity for KSD and positive genetic testing (58.3% of patients with causative variants were determined to have a positive family history). However, the limited cohort of patients from a highly specialized stone center with scarce ethnic variability was an important limitation.

Targeted gene panel sequencing and WES began to be widely used in clinical settings, leading to a substantial growth of the diagnostic yield over the past two years. Schönauer et al. [48] tried a targeted NGS approach, achieving a detection rate of monogenic causes of 6.8%. The authors also identified factors that could predict the likelihood of a positive genetic test result (such as an age at first stone < 40 years, frequent recurrence, mild CKD, and bilateral KSD), suggesting that genetic testing should be considered in the routine work-up of patients with these clinical features. Gefen et al. [49], on the other hand, focused on pediatric patients, finding a positive genetic test in 32% of individuals. Nonetheless, excluding probable (5.4%) and possible (15%) KSD-associated variants, a definite diagnosis with specific pathogenic variants was reached in only 11.5% of cases. Similarly, Mandal et al. [50] identified variants in 20.4% of patients, but considering only patients showing pathogenic and likely pathogenic variants, the total diagnostic yield considerably decreased to 11.1%. Even in the study by Wang et al. [51], the diagnostic rate (46.3%) dropped to 25.6% when excluding patients with uncertain variants according to the American College of Medical Genetics Genomics (ACMG) classification or novel discoveries (not reported in the ClinVar database yet).

Two recent studies on larger pediatric cohorts yielded improved results. Vaisitti et al. [52] analyzed data from 191 pediatric patients affected by kidney diseases who underwent WES, reaching a detection rate of a monogenic disorder in 37.1% of children. Focusing on the KSD subgroup, the diagnostic yield was notably high (45.5%). Their data revealed that over 50% of patients with a positive genetic test had a family history of KSD. These results were strengthened by Liu et al. [53], who collected clinical and genetic data from a Chinese cohort of 218 pediatric patients with KSD who underwent WES. The diagnostic yield achieved was almost 37% of all cases. The authors confirmed the common factors associated with a higher likelihood of a positive genetic test, as previously evaluated in earlier studies.

**Table 1:** Key characteristics of selected studies. NL, nephrolithiasis; NC, nephrocalcinosis; N.A., not available; and WES, whole-exome sequencing.

Author	Population (% Female/Male)	Adult/Pediatric	Nephrolithiasis (%)	Nephrocalcinosis (%)	NL + NC (%)	Age Range (Years)	Age of Onset (Mean)	Family History (%)	Consanguinity (%)	Extrarenal Features (%)	eGFR (Mean)	Genetic Approach
Halbritter et al. [41]	272 (37.1/62.9)	Both	90.0	6.0	4.0	1–81	21.2	N.A.	2.2	N.A.	N.A.	Targeted sequencing
Braun et al. [42]	143 (49.7/50.3)	Pediatric	86.0	11.9	2.1	<18	6.6	N.A.	N.A.	N.A.	N.A.	Targeted sequencing
Daga et al. [43]	65 (50.8/49.2)	Both	49.2	33.8	17.0	<25	3.5	N.A.	N.A.	N.A.	N.A.	WES
Amar et al. [44]	235 (39.2/60.8)	Both	100.0	0	0	1–76	26.7	47.0	53.0	N.A.	N.A.	Targeted sequencing
Fang et al. [45]	82 (N.A.)	Pediatric	N.A.	N.A.	N.A.	<18	4.6	N.A.	N.A.	Neurological (22.4); cardiological (13.0); vision problems (69.4); hearing loss (5.1); and skeletal deformities (4.3)	N.A.	WES
Zhao et al. [46]	105 (33.0/67.0)	Pediatric	100.0	0	0	0.2–11	3.1	18.1	1.9	N.A.	N.A.	WES
Ziyadov et al. [47]	48 (39.6/60.4)	Pediatric	100.0	0	0	1–16	N.A.	58.3	N.A.	N.A.	Low in 8.3%	Targeted sequencing
Schonauer et al. [48]	236 (34.0/66.0)	Adult	100.0	0	0	18–86	34.8	25.0	2.0	Type 2 diabetes mellitus (23.0); hypertension (54.0); and obesity (32.0)	83 mL/min/1.73 m^2^	Targeted sequencing
Gefen et al. [49]	113 (41.0/59.0)	Pediatric	73.5	22.1	4.4	6–16	N.A.	53.0	N.A.	Developmental delay (20.0); prematurity (19.0); failure to thrive (15.0); seizures (7.0); eye abnormalities (3.0); dental abnormalities (2.0); anddeafness (2.0)	None with ESKD	Targeted sequencing
Mandal et al. [50]	54 (40.7/59.3)	Pediatric	100.0	0	0	1–18	5.0	N.A.	N.A.	N.A.	110 mL/min/1.73 m^2^	WES
Wang et al. [51]	82 (32.9/67.1)	Pediatric	100.0	0	0	0.17–15	6.3	N.A.	N.A.	N.A.	N.A.	WES
Vaisitti et al. [52]	22 (45.5/54.5)	Pediatric	45.5	0	54.5	<18	N.A.	36.4	N.A.	N.A.	N.A.	WES
Liu et al. [53]	218 (N.A.)	Pediatric	N.A.	N.A.	N.A.	0.3–13	3.7	N.A.	N.A.	N.A.	109 mL/min/1.73 m^2^	WES

**Table 2:** Number of individual diagnoses divided by included studies. Cys, cystinuria; HC, hypercalciuria; RHUC, renal hypouricemia; dRTA, primary distal renal tubular acidosis; DD/NL1, Dent disease/nephrolithiasis type 1; HHRH, hereditary hypophosophatemic rickets with hypercalciuria; FHHNC, familial hypomagnesemia with hypercalciuria and nephrocalcinosis; HOMG5, hypomagnesemia 5, renal, with ocular involvement; BS, Bartter syndrome; PH1, primary hyperoxaluria 1; LS/DD2, Lowe syndrome/Dent disease 2; aHC, absorptive hypercalciuria; DRTAd, renal tubular acidosis with deafness; PH2, primary hyperoxaluria 2; APRTd, adenine phosphoribosyltransferase deficit; HGly, hyperglycinuria; Xan, xanthinuria; HPP, hypophosphatasia; HPRTd, hypoxanthine guanine phosphoribosyltransferase deficiency; PKHD1, autosomal recessive polycystic kidney disease; PH3, primary hyperoxaluria 3; VDDR2A, vitamin D-dependent rickets, type 2A; NPHLOP1/FS, hypophosphatemic nephrolithiasis/osteoporosis/Fanconi syndrome; and NPHLOP2, hypophosphatemic nephrolithiasis/osteoporosis-2.

Author	Cys	HC	RHUC	dRTA	DD/NL1	HHRH	FHHNC	HOMG5	BS	PH1	LS/DD2	aHC	DRTAd	PH2	APRTd	HGly	Xan	HPP	HPRTd	PKHD1	PH3	VDDR2A	NPHLOP1/FS	NPHLOP2	Total	Diagnostic Yield (%)
Halbritter et al. [41] Total	22	4	4	1	2	1	2			1			1										1	2	41	14.9
Pediatric	9	2	3		2		1			1			1										1	2	22	20.8
Adult	13	2	1	1		1	1																		19	11.4
Braun et al. [42]	3	2		3			1	1	1	1	2	2										2	5	1	24	16.8
Daga et al. [43]	1			1			1	3	3	4				2									6	1	22	29.4
Amar et al. [44] Total	1			3											1								15		20	8.5
Pediatric				2											1								5		8	11.0
Adult	1			1																			10		12	7.5
Fang et al. [45]	5	1							1	9				2							5		1		24	29.3
Zhao et al. [46]	11									9				2							16				38	36.0
Ziyadov et al. [47]	8	3								4						2								1	18	37.5
Schonauer et al. [48]	7			5																				4	16	6.8
Gefen et al. [49]	2					4	1			2								1			3				13	11.5
Mandal et al. [50]	1						1			1							3								6	11.1
Wang et al. [51]	5		1			1	1			4				3			3				3				21	25.6
Vaisitti et al. [52]	6									2										1			1		10	45.5
Liu et al. [53]	14	3		2	1	2	1			21		2		9	1	1			2		21				80	36.7
Total	86	13	5	15	3	8	8	4	5	58	2	4	1	18	2	3	6	1	2	1	48	2	29	9	333	

## 4. Discussion

As a whole, our literature review distinctly shows that there are currently few data on the adult population, with further studies needed to clarify the actual utility of genetic testing in adulthood. In any case, it seems clear that the genetic diagnostic yield in adult KSD patients is considerably low, contrasting with what emerges from studies on the pediatric population.

It is important to consider that a high percentage of patients with a positive genetic test could reach a certain diagnosis only with an accurate clinical and metabolic evaluation, with a remarkable decrease in the genetic diagnostic yield in the remaining cases, both in pediatric and adult populations.

Regarding the more suitable genetic technique, it appears clear that an exome sequencing approach is more advisable, considering the higher diagnostic yield observed compared with the one reached by a targeted gene panel approach. Nonetheless, we should underline that WES may miss some mutations due to the suboptimal coverage of clinically relevant regions, such as introns and promoters. This limitation can be addressed by combining it with CNV assays, which can enhance the rate of causal diagnoses.

Concerning characteristics that may influence the diagnostic yield, we should underline that several factors have already been associated with a higher likelihood of a positive genetic test [54,55], such as a lower age of KSD onset, a familial history of KSD, consanguinity, and extrarenal features (sensorineural hearing defects, ocular abnormalities, neurological disorders, statural growth deficit, and bone disorders). Although the studies considered here associate several clinical factors with a higher risk of a monogenic disorder with a different significance level, we must observe that a considerable percentage of patients with a molecular diagnosis of KSD did not show any of these features. Thus, we cannot rely solely on these factors to determine which patients should be considered for genetic testing, waiting for further studies that would clarify this aspect.

This study has several strengths. First, our systematic review of the currently available literature ensured a relatively large population, with limited bias thanks to the exclusion of studies with extremely selective inclusion criteria. Moreover, our research allowed us to analyze the diagnostic yield of genetic testing in subgroups of clinical interest. On the other hand, the limitations of this study are that most of the included studies enrolled only patients with a clinical suspicion of a genetic form of KSD. Moreover, 6 out of 13 studies (46%) collected data of patients followed-up at tertiary care centers. Nonetheless, when the genetic analysis was performed in a cohort of an adult general population, the frequency of any of 10 Mendelian forms of KSD (not including cystinuria) turned out to be only 3 among 380 patients with more than 40 years of age and a personal history of KSD, corresponding to a prevalence of less than 1% [56]. Furthermore, the results for both pediatric and adult patients might not necessarily apply to the general population of stone formers at large.

## 5. Conclusions

Genetic testing should certainly be considered in pediatric patients with KSD and in selected subgroups of adult patients with suggestive features, such as a positive family history, the presence of CKD, and nephrocalcinosis; it should be performed with an exome sequencing approach for better performance, unless some clinical or biochemical data strongly address a specific genetic disorder that may allow the use of a panel.

The utility and effectiveness of a modern genetic approach are unquestionable considering practical implications, such as the possibility of an earlier diagnosis (reducing the risk of recurrence, complications, and unnecessary radiological as well as biochemical follow-up), familial counseling, and tailored therapy for patients [56,57]. Thanks to the identification of “actionable genes”, it is indeed possible to ensure specific treatments for our patients based on the presence of fine-mapped pathogenic variants [58].

However, we should carefully evaluate its use based on individual characteristics, avoiding it in patients in which a diagnosis is already possible with a meticulous stone examination and an accurate determination of the blood and urine metabolic profiles.

## Data Availability

All data generated or analyzed are included in this article. Further enquiries can be directed to the corresponding author.
